# Why do transgender individuals experience discrimination in healthcare and thereby limited access to healthcare? An interview study exploring the perspective of German transgender individuals

**DOI:** 10.1186/s12939-023-02023-0

**Published:** 2023-10-10

**Authors:** Tobias Skuban-Eiseler, Marcin Orzechowski, Florian Steger

**Affiliations:** 1 Institute of the History, Philosophy and Ethics of Medicine, Faculty of Medicine, Ulm University, Ulm, Germany; 2 kbo-Isar-Amper-Klinikum Region München, München-Haar, Germany

**Keywords:** Transgender individuals, Discrimination, Stigmatization, Access to healthcare

## Abstract

**Background:**

Transgender individuals experience limited access to healthcare. This results not least from experiences of discrimination to which they are exposed in the health system. These contribute to transgender individuals having poorer health than cis individuals, i.e. individuals whose sex assigned at birth is in line with their gender identity. It is an ethical duty to take effective measures to minimize inequalities in medical care. At best, such measures should also be assessed as appropriate from the perspective of those affected in order to be accepted and thus effective. It is therefore important to know whether measures touch on the subjectively assumed reasons for experiences of discrimination. Hence, to be able to take appropriate measures, it is important to identify the reasons that transgender individuals see as causal for their experiences of discrimination in healthcare.

**Methods:**

We conducted semi-structured interviews with 14 German transgender individuals and asked them about their own experiences of discrimination in healthcare and their assumptions on the reasons for discrimination. We analyzed the responses using the method of structured qualitative content analysis.

**Results:**

13 transgender individuals reported experiences of discrimination in healthcare. These emanated from different professional groups and took place in trans-specific as well as general medical settings. We were able to identify a total of 12 reasons that transgender individuals see as causal for their experiences of discrimination: (1) internalized trans-hostility and “protection” of cis individuals, (2) lack of knowledge/uncertainties regarding transition, (3) “protection” of a binary worldview, (4) binary worldview in medicine, (5) structural deficits, (6) asymmetric interactions with specialists, (7) current political debate, (8) view of transgender individuals as a “burden for society”, (9) objectification, (10) homophobia, (11) misogyny/androcentrism and (12) discrimination as reaction to discrimination.

**Conclusions:**

German transgender individuals have a very differentiated picture regarding their subjective reasons for experiencing discrimination in healthcare. Overall, disrespect regarding gender identity and a confrontation with foreignness seems to be seen as the decisive factor. Thus, it is not enough to focus only on measures that aim to remedy the information deficit on the part of medical providers. Measures must be taken that can create a granting and respectful attitude towards transgender individuals.

**Supplementary Information:**

The online version contains supplementary material available at 10.1186/s12939-023-02023-0.

## Background

Transgender Individuals, i.e. individuals whose gender identity differs from their sex recorded at birth, experience significant restrictions regarding access to healthcare [1–4]. This constitutes a particular burden for them, as the access restrictions also affect those medical interventions that are needed in the context of the transition process [5, 6]. This contributes to the fact that transgender individuals in general have a poorer health status than cis individuals, i.e. individuals whose sex assigned at birth is in line with their gender identity [7]. The experience of discrimination in healthcare is a significant factor that impedes access to healthcare for transgender individuals [8, 9]. Negative experiences can make them less likely to seek medical care, thus making them more vulnerable to mental and physical problems [10]. Anticipation of discriminatory experiences or the expectation of being inadequately treated leads transgender individuals to avoid contact with the health system [11–13].

Moreover, minority stress theory [14, 15] can convincingly demonstrate that experiences of discrimination for people who belong to a sexual minority, like transgender individuals, may compromise their mental health. Suffering specific stressors in the wake of stigma, victimization, prejudice, and discrimination, in addition to universal everyday stressors, results in disproportionate overall psychological distress that people do not suffer if they do not belong to a sexual minority [16]. In addition to these group-specific phenomena, it is postulated that stigma-related minority stress can lead to dysfunctional emotion regulation, interpersonal problems, and altered cognitive processes, additionally increasing the risk for developing mental illness [17].

Within the group of transgender individuals, those with a non-binary gender identity are particularly affected by discrimination and limited access to healthcare [18, 19]. Individuals who describe themselves as non-binary have a gender identity that is not exclusively girl/woman or boy/man [20]. These individuals might feel misunderstood about their gender identity even in contacts with health professionals who specialize in treating transgender patients [18]. Non-binary individuals may feel that they are forced to use a binary medical narrative in healthcare interactions [18]. Due to increased experiences of discrimination and stigmatization, non-binary individuals show high levels of depression and anxiety, at times exceeding those of their binary counterparts [21, 22]. In addition, non-binary transgender individuals receive significantly less support from family and friends and experience a higher risk of cyberbullying than cis or binary transgender individuals. Therefore, many non-binary individuals suffer from feelings of isolation and sadness [23].

It is an ethical duty to minimize discrimination and inequalities in medical care that transgender individuals have to endure [24]. Numerous studies deal with possible measures that can be taken to prevent transgender individuals from experiencing discrimination in healthcare. Trans-specific knowledge should be integrated in the education of medical staff; whereby, professionals should be enabled to deal with the special circumstances and health needs of transgender individuals. These educational measures are particularly in demand for the nursing staff, as they often represent the first contact points for patients. Attention to the specific aspects of medical care for transgender individuals should take place already during basic medical training of all health professionals [25–31]. Expanding research on transgender individuals, their needs and mechanisms of discrimination is also seen as a way to reduce discrimination [9, 20, 32]. Political and institutional changes might also prove helpful in reducing discrimination against transgender individuals. In this context, political measures that protect the rights of transgender individuals [25, 32], specialized psychotherapeutic training [33], specific case management teams for the care of transgender individuals [34], and cooperation with the LGBTQI community should be promoted [35]. With regard to health insurance companies, there are calls to cover expenses for specific trans-care [36] and to address trans-care more decisively [37]. A variety of demands concern respect for the special needs of transgender individuals in healthcare [33, 37–42], and the creation of an environment that affirmatively embraces their particular life perspectives [8, 9, 38, 42–46]. These measures differ in character but seem to be primarily aimed at the following goals: (a) the reduction of information deficits on transsexuality, (b) the expansion of specific research on the care of transgender individuals, (c) the closing of legal loopholes and institutional structural deficits and (d) the reduction of inequalities regarding health insurance benefits. As convincing as these measures may sound at first, the question arises whether they are also suitable from the perspective of those affected. Transgender individuals might not regard these measures as having an impact on the subjectively assumed grounds for discrimination. In such case, these efforts could possibly not be able to effectively counteract their experiences of discrimination in healthcare. Furthermore, transgender individuals might not be willing to accept them. Thereby, their efficacy might be decreased. Though the measures could be symptomatically effective, they nonetheless could miss what is at the very core of discrimination. Therefore, it might be useful to inquire transgender individuals about their experiences of discrimination in healthcare, what they assume to be the reasons for being discriminated, and which measures they would consider most urgent to improve this regrettable situation. This can help to select from the multitude of proposed measures those that seem most effective from the perspective of transgender individuals. In this paper, we therefore pursue the following research questions: (1) What experiences of discrimination do transgender individuals have in healthcare today and from whom do these experiences originate? (2) What reasons do transgender individuals see for these experiences of discrimination? (3) From the perspective of transgender individuals, what measures should be taken to minimize discrimination and thus improve access to healthcare?

## Methods

In order to answer these research questions, we have conducted problem-centered semi-structured exploratory interviews with 14 German transgender individuals. As a research method, semi-structured exploratory interviews allow a certain flexibility in the conduct of interviews, with the possibility of asking ad hoc questions in order to concentrate on topics central to the research or to clarify statements of the interviewees in the form of follow-up questions [47, 48]. The aim of such interviews is to gain subjective views of the interviewees on the topic of the investigation with consideration of their background and experiences [49].

Preparation of the interviews proceeded in several steps. First, an extensive review of the scientific literature on the research subject was conducted. This initial research combined with the interdisciplinary background of the project researchers (psychiatry, medicine, medical ethics, philosophy, and political science) led to the definition of focal topics for the interviews. Second, central issues concerning these topics were formulated in the form of questions and detailed interview guidelines were developed (for an English translation of our detailed guidelines see Additional file 1). In the following step, possible interview partners were contacted. The criteria for participation were being of full age and identifying as transgender individual. The initial contact occurred through several German internet networks of transgender individuals through e-mails with an introductory note about the research project. Persons that expressed willingness to participate in the research were informed in detail about the research aim, procedure, possibility to withdraw from the research at any moment, and protection of their personal data. Only individuals that expressed consent for participation were invited for an interview.

The planned procedure for the research was to conduct a minimum of 10 interviews, after which the number of interviews was to be evaluated in light of the information provided by the respondents. Data saturation was then discussed within the team of the researchers. We assessed data saturation based on the additional relevant data that could be collected during the interviews [50], and the number of interviews was extended to 14. The interviews were conducted from February to May 2023 through a secure online communication platform hosted by the university at which the research was conducted. Individual interviews lasted from 31 to 86 min. The interviews were conducted in the native language of the participants by a male researcher with a MD degree and certified as a specialist in psychiatry. The interviewer had substantial knowledge of qualitative research methods, research topic, and had sufficient experience in the conduct of research interviews. No previous relationship with the interviewees had been established prior to the study’s commencement. Each of the interviews was conducted on the basis of the same catalogue of 21 questions.

The interviews were digitally recorded and transcribed. The transcription of the interviews served as a basis for the analysis of the content of the information provided by the interviewees. The analysis was conducted following the procedure of structured qualitative content analysis [48] and thematic analysis [51, 52]. The responses were first reduced to their core elements and statements, manually coded, and extracted from the body of the interviews. In the following step, these elements were systematized through clustering into response topics and subtopics. These topics mirror important recurring themes touched upon during the interviews. For an illustration of the presented themes, representative quotes were translated from German into English. To triangulate the results, the process of the analysis was first conducted by one researcher and then separately verified by a second researcher. Differences in coding and analysis were discussed within the research team. Due to the nature of qualitative research, the results do not aim to provide a representative view of the group under investigation. They rather provide an overview of the subjective statements provided by the sample under investigation. This might be considered a limitation of our study. However, we are convinced, these results allow important insights into the research topic.

## Results

### Participants

We were able to recruit 14 transgender individuals as interviewees. Their age ranged between 18 and 54 years (medium 31.14). Three individuals (21.43%) were born male and 11 (78.57%) female. Eleven individuals (78.57%) identified themselves as transgender men, with two of these also identifying as agender and two also identifying as non-binary. Three (21.43%) identified as transgender women. On average, interviewees had been openly transsexual (outed) to other persons for 8.36 years (range 3–15 years). Their transsexuality was known within the healthcare system for an average of 6.64 years and thus had between 2 and 11 years of experience as transgender individuals with the healthcare system (see Table 1).

**Table 1:** Demographics of interviewed persons

Total number of interviewed transgender individuals		N = 14
Age		18–54 years (medium 31.14 years)
Gender assigned at birth	female	N = 11 (78.57%)
	male	N = 3 (21.43%)
Gender identification	transgender man	N = 7 (50.00%)
	transgender man/agender	N = 2 (14.29%)
	transgender man/non-binary	N = 2 (14.29%)
	transgender woman	N = 3 (21.43%)
Years since outing to any person		3–15 (medium 8.36)
Years since outing to healthcare system		2–11 (medium 6.64)

### Experiences of discrimination

#### General observations

Thirteen individuals reported experiences of discrimination in healthcare. These came from different disciplines and persons and took place in a wide variety of settings. Discrimination by general practitioners and endocrinologists was reported by 11 interviewees, by gynecologists by seven, by other patients and generally in the healthcare system without specifying specific persons by three, by health insurance staff by two, by surgeons, blood donation staff, speech therapists, in psychosomatic hospitals or emergency rooms, or by nursing staff following gender reassignment surgery each by one interviewee. Eleven of our interview partners reported that experiences of discrimination specifically occurred in settings specialized in the treatment of transgender individuals.

#### Misgendering and deadnaming

Eleven individuals reported that they were addressed or referred to in their biological rather than in their perceived gender identity, or that the names assigned to their biological gender continued to be used (deadnaming). Five reported that they had experienced this form of discrimination in trans-specialized settings, e.g. endocrinologists or psychologists specialized in trans-care. For example, one person who also identified as agender, reported to be continuously addressed with pronouns against declared will. Professionals responded that it was *“too inconvenient”* not to use pronouns. Another interviewee was told that omitting pronouns was *“dehumanising”*, which is why a request for pronoun-free address was not granted. One person reported that blood values continued to be interpreted in terms of the gender assigned at birth. This happened regardless of having pointed out that a long-term opposite-sex-hormone-therapy could have caused shifts in the blood values corresponding to the perceived gender identity.

#### Experiences of disregarding specific medical needs of transgender individuals

Ten respondents reported experiences of discrimination through disregarding their specific medical needs as transgender individuals. Six experienced this form of discrimination in trans-specialized settings. One person reported that an endocrinologist *“presumed”* that he was *“deliberately asking for too much testosterone”*. The background was that he had suffered from mood swings and menopausal symptoms. One person reported that a speech therapist had *“insisted”* that the voice should be identified as either clearly male or female, although he had expressed the desire to find a voice fitting to him being also agender. One non-binary person reported it would have been *“presumed”* that the aim of the treatment with testosterone would be to be *“all male”*, although this was not intended by the interviewee at all. One person had not been able to openly disclose being also non-binary to an endocrinologist because of the fear not to receive a prescription for hormonal treatment. Another person reported that a psychologist had classified transsexuality and a non-binary identity as part of mental health problems and refused to provide support for transition until other mental health problems had been *“resolved”*. The psychologist had also recommended to come out to the parents although the interviewee did not want to do that.

#### Compulsion to legitimate one’s transsexuality

Nine individuals reported that they were forced to disclose their transsexuality against their will. Three experienced this form of discrimination in trans-specialized settings. One person mentioned that transgender individuals would often unwillingly end up in the role of an *“enlightener”*. One person reported that it was *“sometimes a bit of a struggle”* to explain one’s own transsexuality to health professionals. Another person expressed the impression that especially the health insurance companies would *“basically doubt”* transsexuality. Likewise, one person mentioned that it was discriminating and degrading to have to legitimize and prove one’s own transsexuality again and again to a psychologist who had to *“judge”* the *“truth”* of transsexuality in the process of transition.

#### Experiences of harassment

Seven individuals reported that they experienced harassment in healthcare. Five reported these experiences having happened in trans-specialized settings. One person reported being *“formally paraded”* to other staff after a mastectomy. Another mentioned being asked about her biological genitalia: this would have been done to *“determine”* whether she was homosexual. Another person was asked about her *“wide hips”*. A psychiatrist had said that he found them *“quite nice”*. The person felt very uncomfortable and powerless because she had to rely on the psychiatrist and his “trans competence”: *“It was a really strong position of power that he had over me, because I couldn’t just go to another doctor”*. Another person reported that she was repeatedly confronted with unpleasant questions about her sexual life in the context of her transition, which had *“nothing to do with transsexuality”*. At the same time, she had felt *“forced”* to answer these questions. One person had been asked to take off her jacket at a psychiatrist’s so that he could *“determine”* how much she would physically “fit” into her perceived gender. Another person had been asked very explicit questions about her sex life by a psychologist. For example, she was asked about her partner’s genitals, and *“whether and how they were sexually active”*.

#### General disrespect regarding gender identity

Six individuals reported that they had felt discriminated due to a general disrespect towards their gender identity. Two had experienced this in trans-specialized settings. For example, one person reported that a psychologist took the position that one should *“accept”* one’s gender assigned at birth and not go down the path of transition. One person (a transgender man) mentioned that a receptionist laughed when he was called as a woman. Another person (a transgender woman) reported that she repeatedly had to hear discriminatory phrases in healthcare such as “*when you were a woman…”*.

#### Experiences of stigmatization

Six individuals reported experiences of stigmatization, with one person having experienced this in a trans-specialized setting. One person had experienced *“strange looks”* from other patients in the waiting room. Another person (a transgender man) had felt *“looked at strangely”* in a gynecology department, which is why he avoided consulting the gynecologists. Thus, after his hysterectomy, he did not attend the necessary follow-up appointments.

#### Refusal of care

Six individuals experienced discrimination through a refusal of care. One person reported having experienced this form of discrimination in trans-specific settings. For example, one general physician refused to treat a person who identified as transgender man because he considered the parallel occurrence of male problems such as hair loss and female problems such as post-menstrual syndrome to be *“too complicated”*. One person (a transgender woman) had been refused treatment by gynecologists several times because she was *“on paper a man”*: *“And then I was treated secretly in the morning so that no one would see me”*. One person had been refused treatment by an endocrinologist. The physician had not been willing to prescribe an individualized hormone regimen for a person also identifying as non-binary. One person was refused follow-up care by a general physician after gender reassignment surgery.

#### Experiences of disrespect of non-binarity/agenderism

Four individuals reported being explicitly discriminated against because their non-binarity or agenderism was not respected. Two experienced this form of discrimination in trans-specialized settings. One person experienced *“real rejection of agender identity”*. Another reported that a psychiatrist checked whether *“his own binary image was confirmed”*. One person reported that transgender individuals also identifying as non-binary were *“discriminated against more severely in the health system”*. He therefore felt *“forced”* to act as a trans-man to receive trans-specific treatment, even though he also identified as non-binary. One person stated that an endocrinologist would have *“presumed”* that his transition had been a *“mistake”* after he came out as being also non-binary.

#### Experiences of open unfriendly behavior

Three persons experienced openly unfriendly behavior in healthcare and felt discriminated against as a result. Two experienced this in trans-specialized settings. One person reported about an endocrinologist: *“But he also said […] that he also checks the data a lot when you come with an indication-letter…that it shouldn’t be from Timbuktu or something. And […] he doesn’t let himself be fooled […]”*. Another person reported that she had been very unkindly *“accused”* of being a *“liar”* and of *“falsifying”* her own hormone values.

#### Experiences of objectification

Two individuals experienced discrimination through objectification. This form of discrimination was not reported for trans-specialized settings. For example, one person reported being *“looked at with great interest”* by a surgeon after a mastectomy, which made him feel objectified. Another person reported that after gender reassignment surgery, she went to have stitches removed and then the assistant was called in so that she could “*see those scars”*.

#### Change of experiences of discrimination over time

Four persons reported increased discrimination over the time they had experience in healthcare as transgender individuals. Six felt that they had experienced less discrimination. Three did not notice any change.

### Reasons assumed by transgender individuals for discrimination in healthcare

In the interviews, we were able to identify a total of 12 reasons that transgender individuals believe contribute to them being discriminated against in healthcare (see Table 2). We present them here in decreasing frequency of mention.

**Table 2:** Reasons for discrimination of transgender individuals mentioned by interviewees. The left column names the different reasons, the right column the number of interviewees that mentioned each reason

Internalized trans-hostility and “protection” of cis individuals	N = 10
Lack of knowledge/uncertainties regarding transition	N = 9
“Protection” of a binary worldview	N = 9
Binary worldview in medicine	N = 8
Structural deficits	N = 6
Asymmetric interactions with specialists	N = 5
Current political debate	N = 3
View of transgender individuals as a “burden for society”	N = 3
Objectification	N = 3
Homophobia	N = 3
Misogyny/androcentrism	N = 2
Discrimination as reaction to discrimination	N = 1

#### Internalized trans-hostility and “protection” of cis individuals

Ten individuals regarded internalized trans-hostility and a “protection” of cis individuals as the reason why transgender individuals experience discrimination in healthcare. One person suggested that there might be an implicit impulse to protect transgender individuals from interventions that they might *“regret later”*: *“You want to ‘save’ one cis person and make life difficult for 99 trans people”*. Transsexuality would be seen as *“inferior”*. Another person said that the health system was not able to overcome the social stigma and professionals avoided being stigmatized because of their work with transgender individuals. One person referred to the representation of transsexualism in the media as *“wrong”* because it *“only shows the flashy colorful birds, the strangers, not the less flashy ones who live happily”*. One person saw transsexualism as something that cis individuals could experience as an *“attack on their self-image”*, because contact with transgender individuals would make them question their own gender identity, which could trigger fears. One person suggested that the *“actual goal of the treatment”* was to be *“perceived as a cis individual”*. It would be about *“eliminating”* the phenomenon of transsexuality.

#### Lack of knowledge/uncertainties regarding transition

Nine individuals regarded a lack of knowledge or uncertainty about interventions during transition as a significant reason for discrimination. Several interviewees complained that there was too little teaching regarding trans-specific care and needs. As there would also be too little research activity, one person said, hormones, for example, had to be prescribed off-label, which again could generate uncertainties. One person said that the health system did not actively seek contact with transgender individuals, which was why: *“[…] trans people have to train to be half doctors in order not to be discriminated against and to get the right treatment”*. One person explicitly remarked in this context that she repeatedly overheard that transgender individuals would *“decide”* for transsexuality. *“It is not a decision. The decision was taken from me. It was given by nature”*. Moreover, transgender individuals were excluded from the discourse: *“People prefer to talk about people, not with them”*.

#### “Protection” of a binary worldview

Nine individuals saw a desire to protect a binary worldview as a reason for discrimination. One person suspected that irritations regarding gender identity were severely socially sanctioned because they *“strongly shook”* a binary worldview. Another person said: *“We are attacking gender roles. So even I, as a completely binary trans-man with the most conservative, boring job, am able to launch an attack causing people to question what makes them a man or woman”*. Transsexuality would be seen as a *“trend”*, which was why as a transgender individual one had to *“prove one’s gender identity again and again”*. Another person said it was about *“fear of the strange and unfamiliar”*. Since people did not usually experience their own body as *“inappropriate”*, they were *“not ready”* to accept a confrontation with transgender individuals. According to another person, it was about the *“rejection of the other and different”* because it would *“challenge the status quo, the normal”*. People would be afraid of realizing that *“the foreign might not be so foreign after all”* and *“might have something to do with you”*. If the order created by binarity was threatened, people could *“no longer rely on what they thought before”*, which could *“shift the power relations between men and women”*, which would be socially rejected.

#### Binary worldview in medicine

Eight individuals saw a strong binary worldview as responsible for discrimination in medicine. One person said: *“I often see that non-binary people are simply not taken seriously. So, doctors often have this man-woman-schema, and everything in between doesn’t exist. And if someone is transident, then this person has to change from A to B and not something in between”*. As a result, *“non-binary people are not taken seriously”* and had to *“present themselves as transgender”*. One person particularly noted that the health system adhered very strongly to a binary language. Another person said that healthcare would implicitly differentiate between *“right and wrong”* transgender individuals. Therefore, non-binary people in particular had to *“hide their identity”*. One person suggested that in medicine, the biological aspects of gender were very powerful, which are questioned and challenged by transsexuality. It was, according to another person, the *“fear of the strange and unknown”*: *“One leaves the unknown unknown. […] One leaves one’s prejudices unchanged and also enforces them without contacting the person, asking about it, informing oneself”*.

#### Structural deficits

Six individuals blamed structural deficits for experiences of discrimination. For example, official documents and forms often did not have the option to identify as diverse, transsexualism was still listed as a disease in the diagnostic systems and since diseases are discriminated against anyway, discrimination against transgender individuals was intensified. One person in particular saw the history of medicalization and obligation to treatment as significant. Transgender individuals were *“denied autonomy over their own bodies”* and had to *“foreground their suffering to be accepted as trans”*. According to another person, it was not about *“being allowed to be trans, but about changing one’s transness”*. Another person said that it was the *“forced link”* between transsexualism and suffering that created the feeling that transgender individuals needed to be *“saved”*.

#### Asymmetric interactions with specialists

Five individuals saw asymmetrical interaction with trans-specialists as a significant factor contributing to experiences of discrimination. One person said that trans-specialists tended *“not to talk to trans-persons, but talk down to them”*. Doctors would *“all sit in their ivory tower and […] look at people under a microscope a bit”*. Another person said that it was already discriminatory that cis experts would *“judge”* transgender individuals. One person noted that it was also problematic that only a few institutions explicitly dealt with the treatment of transgender individuals. This led to an undersupply of transgender individuals and strengthened the power position of the few institutions engaged in trans-care. According to another person, it was a form of positive discrimination when health professionals thought there was *“something wrong with trans-people”*, which was why they *“need help”*. Such an attitude would lead to the *“degrading compulsion to undergo therapy”* and also to transgender individuals themselves seeing their gender identity as *“diseased”*.

#### Current political debate

Three individuals blamed the current political debate for experiences of discrimination of transgender individuals in healthcare. According to one person, it was the provocative continuous thematization of transsexuality that ultimately created resistance. It was the *“exaggerated lobbying”* of transgender individuals, through which the problems of society with transsexuality were repeatedly pointed out and what ultimately led to resistance.

#### View of transgender individuals as a “burden for society”

Three individuals felt that there was an attitude of seeing transgender individuals as a “burden for society” and thus encouraging discrimination. One person said: *“I always had the feeling that I was annoying them as a trans-person, that they felt I didn’t really belong in the health system. And why do I come here and want such elaborate therapies when I am physiologically completely healthy?”* Another person mentioned *“society”* might have the impression that transgender individuals could *“negatively impact the lives of cis-individuals because of the costs involved in transition”*.

#### Objectification

Three individuals saw objectification as a mechanism that led to discrimination against transgender individuals. According to one person, objectification made professionals *“not seeing trans-people at eye level”*. Contact with transgender individuals would thus *“oscillate between interest and voyeurism”*. Precisely because transsexuality, according to another person, was *“so foreign”*, cis individuals *“assume the right to direct their attention to it bluntly and inappropriately”*. One person said: *“What also happened quite often was, I don’t know how to put it, but I sometimes call it circus animal syndrome, that is, that you are like a rarity, where everyone comes to look”*. In some cases, according to even another person, one was no longer *“perceived as a human being […] but only as a sexual object”*.

#### Homophobia

Three individuals considered homophobia to be a mechanism leading to discrimination against transgender individuals in healthcare. According to one person, it was an *“inadmissible mixing of issues of sexual orientation with those of transsexuality”*. This particularly affected transgender individuals who experienced themselves as homosexual. One person said that homosexuality and transsexuality were seen *“in the same category”*, which was why internalized homophobia was *“transferred to trans individuals”*. One person said that cis individuals might have the fear *“that you might find someone attractive who should actually be unattractive to you”*.

#### Misogyny/androcentrism

Two individuals mentioned misogyny or androcentrism as reasons for discrimination against transgender individuals. One person suggested there could be an effect of misogyny and trans-hostility *“working together”*. It could be perceived as *“ridiculous […] when a supposedly more privileged person, i.e. a person read as male, puts himself in such a role (female role, author’s note)”*. Another person said it was incomprehensible to society that someone who was biologically born a man would *“want to be a woman”*: *“How can you not want to have this God-given gift of a penis?“* This was also the reason why transgender women were *“presumed”* to be *“men who dress up to abuse women”*.

#### Discrimination as reaction to discrimination

One person named the counter-intuitive fear of being accused of being discriminating as a reason for discrimination against transgender individuals: Because cis individuals would often feel exposed to accusations of discrimination against transgender individuals, there was a *“counter-impulse”*. Because one did *“not want to be seen as discriminating”*, transgender individuals were discriminated against.

### Measures against discrimination in healthcare proposed by interviewees

We were able to identify a total of six areas regarding which transgender individuals saw action as urgent to reduce their experiences of discrimination in healthcare (see Table 3).

Appropriate measures against discrimination as mentioned by interviewees.

**Table 3:** Measures against discrimination of transgender individuals felt to be urgent and appropriate, as mentioned by the interviewees. The left column names the different measures, the right column the number of interviewees that mentioned each measure

Political changes	N = 11
Cooperation with the community	N = 10
Integration of trans-knowledge in education	N = 9
Creating an LGBTQI-positive environment	N = 6
Creation of a granting and tolerant attitude	N = 6
Expanding research on the experiences and needs of transgender individuals	N = 3

#### Political changes

Eleven individuals considered changes at the political level as important. Several interviewees regarded it important to change the guidelines of the transition process. One person mentioned that it would be important to officially approve hormones for trans-treatment so that these would no longer have to be prescribed off-label. In addition, physicians should be freed from the *“undue responsibility”* they had during the transition process. One person called for more rights for transgender individuals so that they could defend themselves if they were discriminated against. Several persons called for the introduction of a self-determination law and for a complete abolition of regulations that currently exist in Germany in the context of the transition process. Several persons considered it important to carry out information- and education-campaigns among the population to intensify awareness for diverse gender identities. Furthermore, cis individuals should be informed about the time consumption by the transition process: *“This is lifetime that no one ever gives back to you”*. In this context, one person mentioned the importance of the participation of cis individuals in anti-discriminatory activities, because transgender individuals were *“the outsiders”*. One person called for the *“integration of trans-bodies into educational documents like books etc.”*.

#### Cooperation with the community

Ten individuals demanded more cooperation with the trans-community. According to one person, sexual minorities should be integrated in the training of medical staff, especially when topics of gender identity and/or orientation are discussed. One person called for the integration of transgender individuals in the discussion of health insurers about criteria for coverage of transition-specific interventions. It was important, another person said, that transgender individuals with whom contact is then maintained *“do not just belong to the colourful birds”* to not further generate prejudices. One person called for more transgender individuals to work in healthcare or for those already working to be open about being trans. One person suggested that certificates should be awarded by the trans-community if someone wanted to offer trans-specific treatments. One person mentioned that the initiative for contacts with the community should come from the transgender individuals themselves: *“I am the minority. It’s simply a matter of numbers. And I can’t just demand from the majority that they all follow my lead. No, I have to approach them”.*

#### Integration of trans-knowledge in education

Nine persons called for the integration of trans-knowledge into medical training. One person called for such training to be mandatory. Several interviewees regarded it important to provide knowledge not only about transsexuality, but also about the impact of discrimination on transgender individuals. Only one person explicitly rejected special trainings, because the group of transgender individuals was *“too small”*.

#### Creating an LGBTQI-positive environment

Six individuals regarded it important to create an LGBTQI-friendly environment in healthcare. One person felt that it was important to change forms so that they did not reflect binarity. It should *“be acknowledged that there are men who can be pregnant”*. One person suggested that a trans-accepting environment was *“more important than hanging a trans-flag”*. Another person suggested that gender identities should be addressed openly and be integrated on institutional home pages. Another person requested that restrooms should always be able to be used in the gender that corresponds to the perceived one.

#### Creation of a granting and tolerant attitude

Six individuals regarded the creation of a granting and tolerant attitude towards transgender individuals as important. Thus, one person mentioned that transgender individuals should be *“trusted with more responsibility for their own bodies”*. Another person called for a reflection of *“sexual norms”*, citing binarity and the fact that *“being a woman is perceived as a deviation from being a man”*. One person regarded it important that professionals were *“motivated to be accepting and benevolent of others”*. Another person demanded that a certain *“overprotection”* be abandoned: transgender individuals should not have to be *“protected from doing something they might regret later”*. Decisions of transgender individuals should be accepted *“even if a medical professional would not have made them that way”*.

#### Expanding research on the experiences and needs of transgender individuals

Three persons called for an expansion of research on the experiences and needs of transgender individuals. Transgender individuals, one person said, should explicitly be included in research regarding pharmaceuticals. In addition, according to another person, an expansion of research could lead to medical professionals gaining more confidence in dealing with transgender individuals.

## Discussion

In this study, we explored a restricted access to healthcare for transgender individuals as a result of being discriminated in the health system. We were particularly interested in identifying the subjectively effective reasons that transgender individuals hold responsible for their experiences of discrimination in healthcare. By that, we intend to contribute to finding effective methods to prevent transgender individuals from experiencing discrimination and thus to improve their access to healthcare.

### Experiences of discrimination of the interviewees

Amongst the transgender individuals we studied, 13 persons reported that they had experienced discrimination in healthcare. That transgender individuals experience discrimination in healthcare and that this can arise from a variety of different professionals and patients is unfortunately not a new finding [8, 9]. However, it is noteworthy that 11 individuals reported that these experiences of discrimination emanated from those professionals who worked in trans-specialized settings. With regard to trans-specialists, intuitively one would possibly assume that they are sensitized to discrimination experiences of their target group and are rather less likely to be the source of discrimination.

However, the opposite seems to be the case. On closer inspection, this finding is not surprising. Especially in the transition process, trans-specialists have a dominant position over transgender individuals. They decide on the issuing of indication-letters for gender reassignment procedures, on the access to trans-specific interventions, on the amount and type of hormones prescribed, etc. Transsexuality may also be much more of a topic of conversation when seeing a trans-specialist than when accessing another professional. In particular, if the trans-specialist is cis, a sense of group belonging to a cis- versus a trans-identical group might become effective. This could cause discrimination of transgender individuals by various mechanisms: (1) An “ingroup bias” could be important, by which persons tend very strongly to regard their own group as superior [52]. Thereby, self-esteem of one’s own group, i.e. that of the trans-specialists, is increased [54]. (2) In addition, cis individuals strongly tend to value their own group more and to discriminate against transgender individuals [55]. (3) Moreover, contacts with trans-specialists could be regarded as taking place in two different forms of social interaction, namely interaction in a system of differentiation and a system of equality. This distinction was introduced into discrimination research by Fibbi et al. [56]. Systems of differentiation are those in which during contacts a decision on access to certain goods is made. In systems of equality all people are to be treated as equally as possible. While in the former a higher rate of direct discrimination is conceivable, in the latter one would rather find forms of indirect discrimination [56]. Although contacts with trans-specialists take place in the healthcare system, which is a system of equality [56], the granting of complex and costly trans-specific interventions is about access to limited goods, so that these contacts also qualify for a system of differentiation. Thus, both direct and indirect forms of discrimination could be effective.

Our interviewees mentioned numerous different ways in which they experienced discrimination in healthcare. These took place in a wide variety of healthcare settings. A similar finding has been made previously [57]. Seven individuals reported either no change in perceived discrimination or even an increase over time. Discrimination against transgender individuals is a significant factor that can negatively impact the health of transgender individuals. As a result, transgender individuals experience more anxiety, depression, psychological distress, eating disorders, substance use, self-injury, suicidality, and suicide attempts [21, 57–62]. Moreover, experiences of discrimination lead transgender individuals to not seek out the healthcare system or to seek it out too late [63, 64]. It is an ethical duty to combat discrimination against transgender individuals in healthcare and thus provide them with unrestricted access to healthcare. This corresponds not least to the Universal Declaration of Human Rights [65] and thus to an ethical cornerstone of our society.

### Reasons assumed by transgender individuals for discrimination in healthcare

To our knowledge, this is the first study in which transgender individuals themselves were asked about what they thought were the reasons for their experiences of discrimination in healthcare. The participants were very differentiated about this topic. We were able to identify a total of 12 different reasons. The effectiveness of a binary worldview was strongly emphasized by many interviewees. This, they said, continued to be strongly effective in society and medicine, including a non-inclusive environment in healthcare, and was strongly defended by cis individuals, among other aspects to save society the high costs of trans-care. Ignorance regarding transsexuality was a significant factor, but trans-specialists in particular tended to treat transgender individuals as non-equals. In addition, there were other effective tools of discrimination, such as homophobia and misogyny, as well as an androcentric organized society. Moreover, transgender individuals regarded themselves as victims of objectification and the current political debate. Lastly, it was an accusation of discrimination against cis individuals that would make them ultimately discriminate against transgender individuals. These reasons concern different levels: the social, the institutional and the personal. Interestingly, in the context of some of these reasons, several interviewees mentioned that health professionals may make an experience of foreignness in contacts with transgender individuals. This was seen as significant to the phenomenon of discrimination.

The philosopher Bernhard Waldenfels has dealt intensively with foreignness and experiences of the foreign. He argues that the foreign is “unsettling” per se. It was experienced as threatening to overwhelm oneself and withdrawed from processes of control, e.g. by the impossibility to determine and classify the foreign. One’s own, on the other hand, was never doubted and was the instance, in relation to which the foreign was interpreted [66]. However, the foreign is also part of ourselves [66], and threatens from within us. It has already been discussed that contact with transgender individuals can lead to questioning one’s own gender identity [67], and thus possibly makes the foreignness in us visible. As this foreignness might be experienced as threatening, processes of defence against this foreignness become more present. This might be a significant mechanism that contributes to discrimination against transgender individuals in healthcare.

However, a lack of information, which was still seen by nine interviewees as a reason for experiences of discrimination, cannot alone explain this experience of foreignness. There are a lot of things in this world about which we do not have a lot of information, but which are not “foreign” in that sense. In our social contacts we might daily meet completely new persons which we never would regard as “foreign”. According to Waldenfels, it is not the lack of information that creates a feeling of foreignness. In the context of transgender individuals, it might be the ambivalent experience of being affected by a diverse uncanny gender identity and suspecting that this might have something to do with oneself [68].

### Measures against discrimination in healthcare proposed by transgender individuals

Our interviewees suggested several measures to improve experiences of discrimination in healthcare. Eleven persons called for political changes to improve the societal situation of transgender individuals, ten saw collaboration with the community as significant, nine called for the integration of trans-knowledge into education, six for creating an LGBTQI-friendly environment in healthcare, six for creating a more granting and accepting attitude toward transgender individuals, and three for expanding research on trans-specific issues. This is broadly in line with measures that have also been proposed to date to reduce experiences of discrimination by transgender individuals in healthcare. These also include calls for societal change [25, 32], collaboration with the community [35], more education [25–31], LGBTQI-friendly environments [8, 9, 38, 42–46], and more research on trans-specific issues [9, 20, 32].

Beyond this, however, our interviewees specifically addressed the creation of a granting and accepting attitude of healthcare providers towards transgender individuals. This raises the question of the extent to which societal changes, contact with transgender individuals, education, an LGBTQI-friendly environment, and more research activities can help to effectively change health professionals’ attitudes. However, if at least a majority of the other changes that our interviewees called for were implemented, we believe that a big step would already have been taken.

### Limitations

There are some limitations that need to be mentioned. First, we only conducted interviews with a relatively small number of people who all originated from Germany. Thus, the results cannot easily be generalized. In addition, the interviewees were disparate with respect to several characteristics: their age, their experiences with healthcare, the nature of their transsexuality, or their gender identity. Moreover, the (biological) gender ratio was shifted towards women. However, we believe that these limitations do not mean that the results cannot be significant. We succeeded in investigating a very broad field of realities in the life of German transgender individuals. Moreover, despite their diversity, the assessments of the interviewees were not very far apart and also not very far from what is already known in the relevant literature. This suggests that the findings may be consistent with the experiences of many transgender individuals. Further research would be needed to establish this beyond doubt. Since there is evidence that discrimination differs between trans men and trans women [69], follow-up studies would be useful to investigate whether the subjectively assumed reasons for discrimination also differ. Furthermore, the method of thematic analysis itself has limitations: the coding system is subjective and thus subject to interpretation. Moreover, only the occurrence of certain themes is identified and not the relationships between the identified themes. Nevertheless, thematic analysis has the advantage of broadly identifying many different aspects, which can be a favorable starting point for further research. Additionally, it should be noted that we did not apply specific intercoder reliability measures. However, when conflicts in data interpretation arose, we discussed them in detail within the research group to ensure that data interpretation was as consistent as possible. Small differences in data interpretation between researchers cannot be ruled out. However, this did in our view not lead to significant data bias. Finally, the male gender of the interviewer should be mentioned as a limitation. The gender of the interviewer can influence the result of the interview [70, 71]. This may have had an influence on the response behavior of the interviewees. However, having all interviews conducted by one researcher offered us the advantage that the interviews could be as standardized as possible. However, the results of this study must be seen in the light of the limitations mentioned above.

## Conclusions

Transgender individuals in Germany continue to experience significant discrimination in healthcare and thus limited access to healthcare. Trans-specialized settings in particular are no exception. Effective measures are needed to remedy this situation. Our interviewees identified internalized trans-hostility, knowledge deficits and a pronounced binary worldview in medicine as the main causes. The confrontation with foreignness in contact with transgender individuals could be a major cause for the experiences of discrimination as this could also make foreignness perceptible in cis individuals. This means that it is not only about providing knowledge about transsexuality if this form of discrimination in healthcare is to be minimized. Rather, it is about changing personal attitudes of health professionals, possibly by reflecting on own gender identity(ies). One possible way to achieve this should be the integration of transgender individuals if measures are to be implemented to reduce their discrimination.

### Electronic supplementary material

Below is the link to the electronic supplementary material.

Supplementary Material 1

## Data Availability

The datasets generated and/or analyzed during the current study are not publicly available due to reasons of sensitivity, but are available from the corresponding author on reasonable request.

## Abbreviations

LGBTQI: Lesbian, gay, bisexual, transgender, queer, intersexual.
