# Supplementary material for: Why do transgender individuals experience discrimination in healthcare and thereby limited access to healthcare? An interview study exploring the perspective of German transgender individuals
Source: Int J Equity Health. 2023 Oct 10;22:211. doi: 10.1186/s12939-023-02023-0 (PMC10566060; doi:10.1186/s12939-023-02023-0)
Supplement: Supplementary file 1 — Supplementary Material 1 [file 12939_2023_2023_MOESM1_ESM.pdf]

Supplementary Material:

Interview Guidelines

Study: Why do transgender individuals experience discrimination in healthcare and thereby limited access to healthcare?

1. **Introductory questions**
    a. In order to address you correctly, I would like to ask you by which (preferred) name and pronouns I may address you?
    b. And may I also ask you your age?
    c. Do you remember the first time you came out to someone as trans?
    d. How many years have you experienced the healthcare system as a trans individual?

2. **Own experiences of discrimination**
    a. Have you had any experiences in the healthcare system in which you were discriminated against or stigmatized?
    b. Can you tell us about these experiences?
    c. Who did the experiences come from?
    d. Were you discriminated against very directly or did you experience indirect discrimination, e.g., people in the waiting room sitting down away, etc.?
    e. Did these experiences take place when you sought trans-specific health services or when you sought health services regardless of your trans identity?
    f. In the time that you have had experiences with the healthcare system as a trans individual, has the amount of discrimination and stigma you face in the healthcare system changed? Have there been improvements or deteriorations? Has the nature of the discrimination changed?

3. **Reasons of discrimination**
    a. What do you think are the reasons or causes for you as a trans individual experiencing discrimination and stigma in the healthcare system?
    b. Could you hierarchize your reasons? In your opinion/experience, what is the most important/significant reason?
    c. Do you think that the reasons you mentioned are specific to Germany or do you think that the reasons you mentioned are those due to which, in an international context, trans individuals experience discrimination in the healthcare system?

4. **Measures against discrimination**
    a. What do you think would be appropriate measures to be taken to protect trans individuals from experiences of discrimination and stigmatization in the healthcare system?
        i. at the political-social level?
        ii. at the institutional level?
        iii. on a personal level?
    b. Which of the measures you mentioned would you consider the most important in protecting trans individuals from experiencing discrimination in the healthcare system?
    c. How realistic do you think it is that the measures you have mentioned will be implemented in Germany?

Is there anything else you would like to share? Perhaps you have suggestions for further measures and/or during our conversation you thought of another discrimination experience?
